# Revealing ‘Eha: A Qualitative Project on Historical Trauma Experiences Among Wāhine

**DOI:** 10.3390/bs14121238

**Published:** 2024-12-23

**Authors:** Samantha Keaulana, LeShay Keli’iholokai, Riko Lee, Pahonu Coleman, Malia L. Kipapa, Ilima Ho-Lastimosa, Jane J. Chung-Do

**Affiliations:** 1 Office of Public Health Studies, University of Hawai’i at Mānoa, Honolulu, HI 96822, USA; riholee@hawaii.edu (R.L.); ho888@hawaii.edu (I.H.-L.); chungjae@hawaii.edu (J.J.C.-D.); 2 Ke Kula Nui O Waimānalo, Waimānalo, HI 96795, USA; leshay@kekulanuiowaimanalo.org (L.K.); pahonu@kekulanuiowaimanalo.org (P.C.); 3 ‘Ohana Kipapa, Kailua Kona, HI 96740, USA; makipapa@ksbe.edu

**Keywords:** historical trauma, Indigenous health, women, Native Hawaiian, trauma-informed care, violence, community-engaged research, colonialism, Indigenous

## Abstract

Historical trauma has been established as a determinant of health among all Hawaiians, but limited research exists on how Wāhine (Native Hawaiian women) uniquely experience historical trauma. A phenomenological qualitative study was conducted to primarily understand how historical trauma, trauma response, and the transmission and modes of intergenerational trauma intersect with sexism and patriarchy among contemporary Wāhine, as described in the Historical Trauma Conceptual Model. With partnership and approval of the Waimānalo Pono Research Hui, interviews were conducted with 13 Wāhine from various generations in Hawai’i. The structural, institutional, interpersonal, and internal levels of ‘Eha (loosely translated as hurt/suffering/to inflict pain/cause hurt or suffering) were generated as prominent themes from the data. Findings from this project communicate the urgency for change to heal Wāhine with radical aloha and to support them in reimagining a world that is inclusive of their needs.

## 1. Introduction

In pre-Western-contact Hawai’i, all genders were revered, including Wāhine (Native Hawaiian women), who were highly respected for their regenerational, procreative mana (power) [1]. Wāhine mana was yielded through sexual and political power; therefore, Wāhine were considered to be of equal position to other genders, including Kāne (Native Hawaiian men) and occupied roles in Hawaiian society that protected their mana. Their sexual and reproductive power allowed Wāhine to extend their genealogies, where birthing children from high-ranking genealogical lines increased their rank and their political power [2]. The preservation of their mana was crucial to the stability and function of Hawaiian society, and to the health and well-being of themselves, their families, and the land.

Similar to other Indigenous populations, the political, social, economic, and cultural-value systems of Kānaka Maoli (Native Hawaiians; used interchangeably throughout the article with Hawaiians) were compromised after the forced assimilation and illegal occupation of the Hawaiian Kingdom by white colonial settlers. Hawaiian people suffered from massive depopulation due to Western and foreign disease [3] and were made minorities in their own homeland. Their language and cultural practices were suppressed and banned, and new Western laws and paradigms disrupted the traditional Hawaiian way of life. As a result, the Hawaiian understanding of wholistic health and well-being, where spirituality, the wellness of land, and the wellness of people are interconnected, was devalued compared to Western biomedical notions of health [4,5]. This shift in paradigm eroded the value, status, and mana of Wāhine, facilitating an erosion of their overall well-being that would last for generations.

Wāhine’s experiences through colonization were unique [6,7]. In the late 1800s, forced Western gender norms transformed Wāhine’s position in society from being highly regarded to one inferior to men. They were criminalized for their sexualities and behaviors, and sex trafficked for foreign settlers and visitors [7]. In the early 1900s, after hula (traditional Hawaiian dance) was suppressed, the traditional art form was prostituted to drive the growth of Hawai’i’s tourism industry [8]. Wāhine were objectified and exoticized as the brown-skinned hula girl, welcoming white Americans to set their gaze on Hawaiian women [9], facilitating an environment that heightened the risk of violence against Wāhine. At the intersection of sexism, racism, and structural violence, Wāhine became more susceptible to modes of intergenerational trauma. Today, the violence experienced by Wāhine of the past is ongoing and a major factor in their current health and social statuses. In fact, the University of Hawai’i found in their sexual harassment and gender-based violence survey that female undergraduates and Hawaiian students experience higher rates of sexual harassment, stalking, dating and domestic violence, and non-consensual contact compared to other genders, races and ethnicities on campus [10,11]. Furthermore, Wāhine, like Native American women, are also missing and murdered at disproportionately high rates, highlighting a disturbing pattern of violence and neglect faced by Indigenous women across the United States. In particular, Wāhine represent over a quarter of missing girls in Hawai’i [12] and 43% of sex trafficking cases are of Native Hawaiian girls who are often trafficked in popular tourist destinations, such as in Waikīkī on the island of O’ahu [12,13].

### 1.1. Current Health and Socioeconomic Disparities Among Wāhine

Presently, Wāhine consistently face poorer physical, behavioral, and mental health and socioeconomic outcomes when compared to major ethnicities in Hawai’i. The leading causes of death among Wāhine include cancer, suicide, heart disease, stroke, and homicide [14]. In addition, Wāhine rate themselves as needing to improve their mental health one to two weeks out of every month, which is 67% more than non-Hawaiian women [10]. More alarming is the mental health status of adolescent Wāhine, who report the highest rates of sadness or hopelessness (47%) when compared to non-Hawaiian females (35.4%) and the State of Hawai’i average (37.7%) [10]. In 2015, 42.2% of Wāhine in the 9th grade reported harming themselves compared to 33.4% of non-Hawaiian female 9th graders and 19.1% of 9th grade Kāne adolescents [10].

Additionally, Wāhine encounter social and economic challenges that negatively affect the health of their ‘ohana (family) and keiki (children). The Office of Hawaiian Affairs [10] is concerned that the overrepresentation of Wāhine in the incarcerated population in Hawai’i will have intergenerational repercussions, including intimate partner violence and adverse childhood experiences (ACEs) among their children. In 2010, Wāhine made up 43.7% of the incarcerated population in Hawai’i, but only 18.4% of the State female population [10,15]. They also experience being psychologically abused by their dates/partners, and experience intimate partner violence earlier in their lives (20.6%) compared to other non-Hawaiian women (13.3%) [10]. When it comes to the economic well-being of Wāhine, they are paid 71 cents for every dollar white men are paid and 82 cents of which Kāne are paid [16]. In education, 20.4% of Wāhine have attained bachelor’s degrees or higher compared to 46% of white women in Hawai’i and 33.5% of all women in Hawai’i [16]. Wāhine who become mothers experience the highest rates of infant mortality in Hawai’i, as there are 8 Hawaiian infant deaths per 1000 live births versus 3.5 deaths for white infants in Hawai’i [10]. Moreover, they are one of three racial groups with the highest percentage of reported signs of postpartum depression [17].

### 1.2. Historical Trauma as a Determinant of Wāhine Health

Historical trauma (HT) has been attributed to the negative mental and behavioral health conditions of Indigenous peoples [18,19,20,21]. Brave Heart [22] defined HT as the “cumulative emotional and psychological wounding over the lifespan and across generations, emanating from massive group trauma experiences” (p. 7). HT includes historical unresolved grief and the historical trauma response. Historical unresolved grief describes the shared grief that is passed down intergenerationally and historical trauma response is a reaction to HT that includes mental and behavioral health issues like depression, suicidality, anxiety, substance abuse, etc. While historical trauma is passed down intergenerationally, Indigenous peoples also continue to face racism, discrimination, and ongoing violence that persists as a result of settler colonialism, resulting in higher risk for mental health disorders [23]. The historical unresolved grief shared by contemporary Wāhine and their ancestors coupled with present day adversities may be associated with current health and social disparities.

The Indigenous scholar, Haunani-Kay Trask [24], offered a gendered insight on historical and structural violence on its impact on Wāhine. She coined the concept “double colonization” to describe the experiences of Wāhine who must fight for their own liberation as women while they fight for the liberation of their people and land. In essence, generational trauma and structural violence experienced by Indigenous women are in part due to oppressive patriarchal misogynistic systems that have subjugated Wāhine as being lesser than Kāne [24]. This disregards the values of gender duality and equality that existed in traditional Hawaiian society [3].

While previous literature has explored the experiences and conceptualizations of Indigenous people on historical and intergenerational trauma [21,25,26], little is known about how Wāhine conceptualize and experience historical trauma. An understanding of Wāhine’s experiences in historical trauma can be important in dismantling unresolved trauma and ongoing structural violence. In addition, exploring historical trauma among Wāhine acts as a preliminary step in being able to quantify the phenomenon to be used to determine policies and strategies to promote wellness and prevent violence among Wāhine. The current project was part of a three-study dissertation that employed a mixed-methods approach to understand historical trauma among Wāhine and begin to quantify their experiences [27].

### 1.3. Indigenous Feminist Theory and Historical Trauma Conceptual Framework

In this phenomenological qualitative project, Indigenous Feminist Theory (IFT) was central to the development of both the interview guide and the data analysis. IFT informed the research process by critically examining how the intersection of gender roles and colonialism shaped the lived experiences of Wāhine. Central to IFT is the idea that the liberation of Indigenous women is inseparable from the liberation of their people, nations, and lands [28]. The theory posits that the oppression of Indigenous women cannot be fully understood or addressed in isolation from the historical and ongoing effects of colonization, which has disrupted not only gender relations but also the social, political, and cultural fabric of Indigenous societies, regardless of origin [29]. In alignment with Indigenous feminist theorists and advocates, this study aimed to decolonize Indigenous women and others by infiltrating and countering the mainstream narrative told by white settlers and men [28,30].

Additionally, the Historical Trauma Conceptual Model (HTCM) [31], which illustrates the transfer of trauma from historical events to contemporary populations, was applied to also inform the interview guide and data analysis in this project. HTCM encompasses three theoretical frameworks: the psychosocial theory linking physical and psychological stress to social environments; political/economic theory which highlights the inequitable impacts of political, economic, and structural determinants of health; and the social/ecological systems theory, which recognizes the dynamics and interdependencies of the past/present, proximal/distal, and life course factors impacting disease. These theories allowed for a comprehensive, multilayered means of illustrating how historical trauma impact Wāhine.

## 2. Materials and Methods

### 2.1. Research Design

A phenomenological qualitative research design was employed to allow for a deep understanding of Wāhine’s perceptions, perspectives, and understandings of the historical and intergenerational trauma phenomenon [27,32]. Phenomenology concerns the first-person point of view of lived experience and how participants make meaning of that experience, which results in a rich description of the essence of a phenomenon [33]. Phenomenology, however, has Eurocentric and colonial roots, and often conceptualizes the human experience through an individual lens and without considering the historical, social, and political conditions that shape experiences [34]. In this project, phenomenology was employed with a community-engaged approach to decolonize the process of the research design. Furthermore, results of the study were analyzed wholistically to consider the historical, social, and political implications of the findings.

This project was built upon an established, long-term relationship between the lead researcher and the Waimānalo Pono Research Hui (WPRH). WPRH is an academic and community partnership between residents of Waimānalo and academic researchers [35,36]. WPRH served as the community ethics and advisory board for this project because they possess a set of pono (just/righteous) research principles, which provides a code of conduct for researchers who wish to engage and deploy projects in Waimānalo [35,36]. WPRH is situated in a predominantly Hawaiian community with experience in various academic and community research projects, as well as community building and mālama ‘āina (to take care of land) initiatives. Prior to the project conceptualization, the lead researcher had a long-standing relationship with community members of Waimānalo and was an active member of WPRH. WPRH bore the infrastructure through their pono research principles that allowed for the Indigenous critical review and approval of this sensitive project. Their protocols on data ownership, researcher accountability, and dissemination in particular were a crucial part of the process to ensure that the research project was respectful of participants, empowered Wāhine, redressed power imbalances between the researcher and the community/participants, and promoted social justice.

### 2.2. Positionality and Research Team

The lead researcher and the majority of the researchers are Kānaka Maoli and Indigenous (Keaulana, Keliiholokai, Coleman, Kipapa, and Ho-Lastimosa). All researchers are deeply affected by the enduring legacies of colonialism and the systems that perpetuate the oppression of Kānaka Maoli and Wāhine. This research is personally significant to the lead researcher, as she seeks to address the hihia (issues) that her kumu (teacher), Aunty Lynette Paglinawan, identified as vital for achieving optimal Hawaiian health. The research team, comprised mainly of Wāhine from WPRH, share a background in social work, counseling, public health, and traditional healing. Their formal trauma-informed training and personal experiences played a crucial role in shaping the project’s design, as well as the conducting and analysis of interviews and sharing of the findings. Lastly, the research team prioritized Hawaiian epistemologies throughout the project and within this discourse.

### 2.3. Participant Group

This project conducted key informant interviews (*n* = 13) to explore Wāhine historical trauma in depth. Purposive sampling was used to select participants who had already built trust with the research team, creating a safe environment for discussing such a sensitive topic. Recruitment strategies included outreach via email, word-of-mouth, and face-to-face interactions. Interviews took place in 2020 and 2021. To participate, individuals had to be at least 18 years old, of Hawaiian descent, and identify as Wāhine.

### 2.4. Data Collection

During the recruitment process, all potential participants were provided with interview questions, a consent form, and a de-escalation form prior to scheduling interviews to clarify the project’s scope and ensure risk mitigation. The de-escalation form was included as community partners and the research team agreed that they needed a safety protocol to collect sensitive information about trauma. As a result, the research team operated from a trauma-informed care framework, which recognizes how trauma symptoms can affect health outcomes and aims to prevent re-traumatization during treatment and care delivery [37,38]. The Crisis Prevention Institute’s De-Escalation Preference Form [39] was selected as it is an established tool to equip professionals with strategies for managing and preventing agitation and it emphasized the importance of understanding nonverbal communication in trauma contexts. The form was shared with and approved by community research partners. Upon scheduling each interview, the form was explained over email, in-person, or by phone to each participant and was utilized to create individualized action plans in the event of a potential crisis. Prior to the start of each interview, the form was shared again, and consent was received with the consent form.

Thirteen semi-structured key-informant interviews were conducted by the lead and secondary researchers in 2020 and 2021. Two researchers were present at each session. The lead researcher facilitated the interview, and the second researcher took notes and helped with probing questions. Prior to the first question, the researchers explained the interview’s purpose and the concept of historical trauma. To ensure clarity, participants were asked to share their personal definitions of historical trauma and to use these definitions throughout the interview. In addition, both researchers briefly shared their genealogies and connections, lived experience, and interest in the research topic.

All participants provided informed consent and were given the option to remain confidential or to be recognized for their contributions. They were also informed that they could change their preference at any point during or after the interview; however, none chose to be identified. The interviews lasted between 1.5 and 2 h and were conducted via Zoom in compliance with COVID-19 protocols mandated by the University of Hawai’i Institutional Review Board. All participants consented to audio recording, enabling verbatim transcription of the interviews. To acknowledge their time and contributions, each participant received a USD 50 makana (gift).

Lastly, the lead researcher followed up with participants the day after each interview to assess their well-being and address any concerns they might have regarding the interview process. This follow-up not only ensured participants’ comfort but also reflected the lead researcher’s commitment to maintaining a strong, supportive relationship with each individual, recognizing them as more than just research subjects. While the lead research expressed gratitude, participants also expressed gratitude by thanking the lead researcher for allowing them to express their stories and working to address historical trauma.

### 2.5. Measures

Interview questions were primarily formulated based on the existing literature on Indigenous Feminist Theory and the Historical Trauma Conceptual Model. The interview guide was shaped by four domains of interest: mo’okūauhau (genealogy), perceived historical loss, trauma response, and intergenerational trauma experiences. Interviews explored historical and contemporary events, experiences, and roles that may have impacted participants. The semi-structured interview guide was developed by the research team and approved by WPRH community members. Table 1 specifies the domains of interest and related interview questions.

#### 2.5.1. Mo’okūauhau Domain

For the mo’okūauhau domain, both participants and the research team shared a story of their genealogy. The majority of participants shared where they are from or who their parents are. Mo’okūauhau (geneaology) is significant in the Indigenous understanding of how the past guides the future, and, in practice, it enables people to recognize and forge connections with each other. The significance of mo’okūauhau is evident in a Hawaiian proverb “i ka wa mamua, ka wa mahope”, which translates to “the future is in the past” [40]. Beginning with mo’okūauhau posed a salient question that helped prime participants for subsequent sensitive questions about their experiences of land and cultural loss [40]. In addition, mo’okūauhau built further pilina (relationship/connection) between the participants and the researchers [41].

#### 2.5.2. Perceived Historical Loss Domain

Historical trauma has been measured with perceived historical loss [20]. Physical and psychological violence, displacement, and cultural dispossession are historical losses that contribute to the loss of land, culture, etc., which impact Indigenous peoples’ health [31]. Questions were formulated to understand how salient historical losses were perceived among contemporary Wāhine. To probe, yet avoid biasing responses, the research team briefly shared an example of Native Americans and First Nations peoples’ experiences with boarding schools.

#### 2.5.3. Trauma Response Domain

The question formulated for the trauma response domain was concerned with how historical trauma impacted them or the Wāhine in their families. In this domain, we probed with social and psychological responses described in the Historical Trauma Conceptual Model (e.g., “has anyone ever experienced substance abuse, domestic violence, depression, etc.”).

#### 2.5.4. Intergenerational Trauma Transmission and Experience Domain

Questions in this domain sought to understand how Wāhine see historical and intergenerational trauma passed down to them or other Wāhine they know. Participants were asked gender-specific questions related to trauma transmission. 

### 2.6. Data Analysis

Transcriptions were analyzed using thematic analysis, an iterative process that generates themes important to the description of the phenomenon and requires researchers to be reflective and reflexive [33,42,43]. Therefore, the transcripts were divided evenly among the lead researcher and secondary researchers and were initially coded individually. A priori codes were created based on the domains utilized to develop the interview questions and were pre-determined by the research team before initial coding. During initial coding, researchers also created a posteriori codes based on emerging codes from the data. Next, the research team met to create a codebook, and the remaining transcripts were coded together. NVivo was utilized for coding in this phase to assist in finalizing themes.

## 3. Results

A total of 13 participants were recruited and interviewed for this project. All participants were women of Native Hawaiian descent. Ages ranged from 18 to 70 years old and most of the interviewees had children. All the participants resided on O’ahu, Maui, or Hawai’i Island. A summary of the participant characteristics is provided in Table 2.

The research team identified violence as a salient theme throughout the transcription review. Participants conceptualized historical trauma with various stories of violence within systems, interpersonal relationships, and within themselves. The violence inherited and passed on demonstrated that individual behaviors were/are not at fault for intergenerational trauma transmission. This also illustrated how historical violence is perpetuated systemically and is often beyond individual control. If individuals are not given or kept from the tools and resources for healing, violence persists. Furthermore, after careful analysis of the data, participants’ experiences demonstrated that historical trauma is not historical as violence committed against Hawaiians remains prevalent. Therefore, historical trauma does not happen in a linear fashion as violence is compounded throughout the generations. The lead author consulted with a community cultural advisor about these findings and determined that findings reflected more about ‘eha than historical trauma. ‘Eha, for purposes of this project, loosely translates to hurt and suffering or to hurt and cause suffering. The lead researcher and cultural advisor ruminated on the study findings together. After discussion on the definition and kaona (hidden meaning) of ‘eha, they decided that ‘eha, or violence, was a better representation of participants’ stories than historical trauma.

To organize the themes of this project, the research team utilized Camara Phyllis Jones’s Levels of Racism theory [44] and the Multilevel Racism and Native Hawaiian Health [45] modeled after Levels of Racism, as they indicate how a construct and experience like racism permeates throughout various levels of society. As a result, four major themes were identified: (1) structural ‘eha, (2) institutional ‘eha, (3) personally mediated ‘eha, and (4) internalized ‘eha. Similar to the Levels of Racism theory, ‘eha manifested at different levels that were interconnected as they interacted and influenced one another. Each theme is described in greater detail below. 

### 3.1. Theme 1: Structural ‘Eha

Structural ‘eha depicts violence that happened historically and was/is reinforced culturally, socially, and psychologically, thereby producing and perpetuating harm and inequality. It is ingrained in society and becomes a part of daily life. As a result, oftentimes it is less visible and maintains unequal power differentials that impact unequal structures [45]. Just as intergenerational trauma is described in Sottero’s Historical Trauma model [31], participants shared how their kūpuna acquired ‘eha through colonialism and settlerism, which was then inherited by the participants and their family members. Participants commonly brought up historical events that forced assimilation and introduced various policies and standards of living that are beneficial to settlers but marginalizing Hawaiians. They often mentioned the introduction of Western worldviews and religion, which brought cultural and social implications among participants, such as the disregard of Hawaiian culture and sacred lands, changed roles and expectations on genders, and led to stigmatization of Hawaiian people and discrimination in their interactions within various systems.

#### 3.1.1. Disregard for Hawaiian Culture and Sacred Lands

Participants commonly conveyed that two historical events, the overthrow of the Hawaiian Kingdom and the extraction of Hawaiianness in Hawai’i (e.g., banning of ‘ōlelo Hawai’i), were culprits of their disconnection with Hawaiian culture and the disregard of Hawaiian practices among non-Hawaiians, tourists, and non-native residents as well as Hawaiians themselves. As a result, Hawaiian culture became inaccessible to participants and/or their kūpuna. For example, the suppression of ‘ōlelo Hawai’i (Hawaiian language) impacted many participants and their family members. Many attributed their loss of cultural identity through the inability of their kūpuna and themselves to speak their native tongue, and therefore, their ability to live authentically and proudly as Hawaiians. Similar to First Nations and Native Americans forced into boarding schools to “kill the Indian”, the loss of participants’ ability to speak ‘ōlelo Hawai’i in Hawai’i’s school system led to more than the loss of language. Participants describe losing their understanding of their genealogies and ancestral stories, exacerbating their disconnection with their culture and, in some cases, their families and communities.

The disconnection through the blatant settler annelation of Hawaiian culture blurred important Hawaiian values like kuleana (responsibility) and kapu (sacredness) for participants. For example, a participant spoke about the disregard of kapu when she witnessed a non-Hawaiian researcher conducting a research project on Hawaiian ‘iwi (bones). While it did not sit well with her na‘au (guts, innards, gut feeling), she shared how research on ‘iwi had become a common practice at the time as there were no laws to protect burials and bones. She stated, “*you take something that’s so sacred, and you… you just wish it up into just material… I felt like this was wrong, really wrong…*”.

#### 3.1.2. Roles and Expectations on Gender

In regard to gender, many mākua and ‘ōpio participants were able to identify the roles and expectations of the previous generations before them and articulate the loss in mana among Wāhine. To illustrate, participants described how their grandmothers’ roles in post-Western-contact Hawai’i were limited to Western domestic roles that were regarded with less power and respect. “*My grandma was only good to watch her kids, cook food… that type of stuff… and I think it was hard for me because I saw her in a whole different light… She was the matriarch of our family and she possessed this ‘ike [knowledge] and this experience that was no longer talked about*”. Many participants believed that religious institutions that replaced the traditional Hawaiian way of life and value systems forced Western gender norms upon both Kāne and Wāhine. Christianity was wielded as a tool in post-Western-contact Hawai’i to colonize and diminish Hawaiian culture and missionary wives made it their mission to transform Wāhine characteristics and behaviors to those accepted by Western standards [46,47]. To do so, they demonized and attacked Hawaiian culture, a culture which upheld the mana of Wāhine and gave them autonomy within the Hawaiian social system [4,46]. In this research project, participants shared that religion still presents limitations for contemporary Wāhine as many believed that Kāne utilized teachings of the Bible to push coverture. “*In the Bible, it says that men are over women. I think it makes men feel like they’re superior… and that they’re smarter and stronger… which happens a lot in very religious households… and it doesn’t matter how men treat you because the Bible says so. Even growing up, my own brothers expected me to do things for them and call me out of my name*”. 

In addition, participants identified that Kāne also took on new roles with Western influence. They recognized that Kāne also suffered from the drastic changes in restrictive gender roles. Such new gender roles were described to have adverse effects on families. Thus, Wāhine began to manage a lot of decisions on behalf of their families and communities, without receiving recognition and being seen as inferior to Kāne. “*I just feel like our Hawaiian Kāne overall, kind of just take more of a… backseat role. And they don’t come to the forefront as much as wāhine. And I feel like there’s something in the, you know, what happened to our people that caused that… our kāne were stripped of their strength and their place in the family, and even in the community. So there’s something like psychological that happened, you know, when our government got overthrown, you know, when there was loss of lives and loss of language and health issues and financial issues*”. Overall, participants expressed frustration with their intimate partners who were Kāne, yearning for a partner who embodied a balanced masculine and feminine energy to strengthen their family. Participants also mentioned that Kāne who were in touch with their culture were better leaders and examples for their children.

#### 3.1.3. Stigmatization and Discrimination

Participants shared how disconnection from Hawaiian culture happened when their Hawaiianness became inferior to other identities in Hawai’i. Hawaiians were stigmatized as lazy or stupid, resulting in structural implications that directly impacted participants’ educational attainment, health, socioeconomic status, and experiences with various institutions. For example, one participant pointed out that Asian kids in her high school were seen as high achieving, and therefore, they tended to receive more guidance and counseling, while she did not receive the same resources until she proved that she had earned it. “*in high school… Japanese and Filipinos were the majority. [They were] all the privileged ones… an example I can share was during my junior year, I noticed they elevated the Japanese and the Filipinos a lot more because the leadership portrayed them as though they’re supposed to be smarter through academics… it wasn’t till I took the initiative, and demonstrated by doing…. I planned and coordinated in my Senior year the grad project for our senior class… And then it was only through my efforts and contribution that the guidance counselor saw my potential and looked beyond the color of my skin, beyond me as one Hawaiian. I guess it was like a rite of passage to prove myself…. because of the success of that class event, it was only then I was treated with sincere respect and given the courtesy of access to scholarship applications and college application waivers for college*”. Structurally, Asian settlers have bolstered power over time in Hawai’i, utilizing the colonial system initiated by white settlers, which facilitated the continual disenfranchisement of Native Hawaiians [48,49].

Overall, the stigmatization and discrimination of Hawaiian people resulted in lack of pride in Hawaiianness, which created harmful intergenerational ripples. “*My grandfather was a big influence over our family… he didn’t want to be known as a Hawaiian. He wanted so much to be American. My grandmother spoke Hawaiian and he told her she cannot do that in the house. It got to the point where he didn’t want to eat Hawaiian food… if I just look at my life and the whole world, I think that’s where I get some of my insecurities*”.

### 3.2. Theme 2: Institutional ‘Eha

Institutional ‘eha was a prominent theme. It refers to ‘eha inhibiting access to power, influencing policy and practices, and limiting access to goods, opportunities, and service. Various institutional ‘eha were described by participants, including the inability of kūpuna to learn their own language, land displacement by blood quantum policies on housing, and negative experiences with the justice system. Many also indicated that trauma to Hawaiians is ongoing through institutional decision making. Furthermore, their accounts of unfair treatment reflected being marginalized by systems that were intended to protect or support them, leading to significant negative effects on their mental health and self-esteem. This theme was organized by two subthemes where institutional ‘eha seemed to impact participants the most: language and culture, and land.

#### 3.2.1. Language and Culture

Participants spoke in depth about ‘eha from Western-influenced government institutions inflicted through policies that systematically changed the culture of Hawai’i. As mentioned, they commonly shared details about the suppression of ‘ōlelo Hawai’i. In 1896, the provisional government enacted Act 57 which suppressed ‘ōlelo Hawai’i by making the English language “the medium and basis for all instruction in public and private schools” [50]. The law punished students for using ‘ōlelo Hawai’i in schools, which resulted in traumatic implications for participants’ family members and themselves. In particular, Wāhine suffered as Western beliefs of male superiority suppressed women’s voices in addition to their native tongue. One participant recalled, “*… being punished for speaking Hawaiian or for wanting to learn Hawaiian… it’s made the wāhine in my family… well, it kind of stripped them of their voice*”. Participants shared how the suppression of ‘ōlelo Hawai’i catapulted into a lack of pride in Hawaiian identity among their kūpuna. In addition, it impacted their own Hawaiian identities as others demonstrated their Hawaiianess with ability to speak ‘ōlelo Hawai’i. Moreover, the inability to speak ‘ōlelo Hawai’i exacerbated their access to Hawaiian cultural ways of knowing and living that exist within the language.

#### 3.2.2. Land

Other institutional ‘eha included ideals and policies that displaced Hawaiians from land and changed their interactions with it. The Western concept of land ownership led to land displacement among many participants and resulted in interpersonal and familial violence. Prior to Western contact, Hawaiians maintained a kinship-like relationship with land, but Western influence encouraged Hawai’i’s monarchy to institute the Great Māhele, which redistributed land and introduced Hawaiians to private land ownership [51]. Unfortunately, many participants and their families were placed at a disadvantage with this Western notion of land. One participant described how her great-grandfather lost land through alcoholism and the manipulation of Americans who owned a bar, while also illustrating the implications of Western substances being introduced to Hawai’i. “*My great-grandfather and great-grandmother had a lot of land… like hundreds of acres kind… So, I guess my great-grandfather liked to drink alcohol, but he couldn’t pay off his tab. So, in return, bar owners would have him sign over his property… now we only have one acre left… it’s a one-acre lot and my papa has 12 siblings… there’s no room and it turned family against each other*”.

Another institutional policy salient among participants was the Hawaiian Homes Commission Act of 1920, which was created to rehabilitate Native Hawaiians who had 50% blood or more through a government-sponsored homesteading program and give them economic self-sufficiency through the provision of land [52]. Such an act was promising as many Hawaiians lost access to their ancestral lands through the Great Māhele [53]. However, participants described their ongoing contentious relationship with the Department of Hawaiian Homelands (DHHL), as they lease land to American corporations and organizations for profit, prohibiting Hawaiians from being awarded land or exposing existing residents to environmental dangers. “*For over 5 years, they didn’t improve a heavily commercial use road that we need to pass through to get home [in the homestead]…instead, we [had to pass]… an old sewage plant and petroleum… to get home…. We don’t even know what we are exposed to in the air from all of this going on in our community. And we drive through this constantly. But I guess you become so assimilated to that… because you get so used to seeing it and it becomes normal when it shouldn’t be anymore*”.

In addition, the participants discussed how the act created disdain and pilikia (trouble) among individuals, families, and communities. One participant married into a family with a homestead lease and shared that she could have been added to the lease as she was a quarter Hawaiian, but her husband’s father refused, and her husband did not support or advocate for her. Another participant reported feeling undeserving of having a lease because other Hawaiian families are in dire need of affordable housing. “*You know, it just disheartens me I feel bad I even get one homestead you know? I feel like other families should have it… You know one family that cannot afford… $1200 plus rent for a little apartment… I always told myself I would never get Hawaiian Homes because I wanted to give that to somebody else that needed that opportunity. But the sad part about it was I needed it because I can’t even afford to live in the actual financial state… I had to resort to go homestead*”. The act, although created with good intention, was described in this project as being divisive. “*… it created division and divisiveness amongst families because you know, [the DHHL commission] represents Hawaiian Homes… So, then you get crooked Hawaiians… that is driving this system to their advantage and people are blatantly seeing this in a community, right? So, you get this divisiveness amongst families*”. Militarism and urban development also displaced participants’ families from land. Participants described being unable to access ‘āina and being forced into urban spaces, resulting in a lack of access to natural resources. “*[My grandparents] lived in the [sugar plantation] camp, and then I guess as things progressed after Pearl Harbor… [there was] development of Waipahu… before, they could easily go to Pearl Harbor, and they could fish and there was oysters and all these natural resources that was readily available… it’s more restricted now, the waters aren’t clean, and my grandpa couldn’t go fishing anymore. Military pushed them out*”. One participant shared the impact of urbanization on her grandmother, “*… the natural resources around her were just depleting because of the development… they went from having a yard… to being in an apartment building, basically sinking their assets*”. 

In addition to experiences of displacement from homelands, participants reported how aloha ‘āina movements (e.g., protection of Mauna Kea and Kaho‘olawe) impacted them. Participants felt equally ignited and a sense of ‘eha about aloha ‘āina movements. On one end, aloha ‘āina movements represent a space to proudly be Hawaiian and learn about their culture, however, the ‘eha was the continuous struggle to justify the importance of protecting ‘āina to everyone. One movement mentioned frequently was the protection of Mauna Kea from a thirty-meter telescope (TMT) construction, as it was ongoing during this project and salient in the media [54,55]. Participants shared the disappointment of Hawaiians having to fight for sacred spaces and dealing with passive aggressive comments from settlers in their respective communities. For example, one participant shared her experience with her co-worker. “*… when I first started working with her, she would make comments about the mauna… the president or founder of our [local grocery store] came out and supported TMT… and she started coming out kind of strongly about like seeing social media with Kānaka saying let’s not support [local grocery store]… and she told me,* ‘*how can Hawaiians make like that to [the local grocery store]. That’s not aloha. You guys talk about aloha, but that’s not aloha.* ‘*… and it’s like, you know, [she] was totally missing the point!… I find it odd that she has roots in [a Hawaiian town]… for a long time her family been here… you call this place home. Maybe [she’s] not Kanaka, but so what kind of connection does she have to this place that is pono [just/righteous] to her?*” Another participant stated when talking about Mauna Kea, “*I don’t know if you guys had come across people in conversation and you just getting this vibe that the person just doesn’t want to get it. They don’t want to understand. Yeah, those conversations I’ve had about TMT and the mauna where I felt that… even with people within the DOE [Department of Education]. And it all goes back to our teachers who are educators. And they wonder why our test scores is like, always the same. Always huge gaps. The disconnect!*” Overall, participants felt people who influence institutions in Hawai’i maintain structural ‘eha through their decision-making that are direct threats to Hawaiians through the desecration of land.

### 3.3. Theme 3: Personally Mediated ‘Eha

Personally mediated ‘eha was the third theme. It describes the abilities, motives, and intentions of others according to the participants’ race and/or gender. Like the Levels of Racism Framework, personally mediated ‘eha can be intentional or unintentional and are condoned by societal norms. Such ‘eha maintains structural and institutional racism. This theme was organized into three subthemes that identify the groups who most commonly inflicted ‘eha on the participants.

#### 3.3.1. Kāne

In this theme, participants’ experiences with Kāne were heightened. They described their feelings of inferiority as Wāhine around Kāne who maintain their decision-making power within families, community organizations, and professional spaces. “*… in our community, especially if it’s headed by Kāne… they decide who they let know and who they include or exclude and most of it is Hawaiian women that they’re excluding. [It’s] this vicious cycle of entitlement as Kāne—as Hawaiian Kāne that they can do it all because they’re Hawaiian Kāne and that they have that right*”.

‘Eha distributed by Kāne was emotional, spiritual, and physical and impacted subsequent generations. However, participants identified that such ‘eha came from generations before them through structural and institutional decisions that directly impacted the Kāne in their families. “*… not being able to practice his culture and learn about his culture has made him a very angry Hawaiian… And that’s led to years and years of domestic violence against both my mom and me*. *It made me feel hopeless and really sad because there was nothing that I could do… I didn’t know when the fighting would stop… it was scary… it sucked*”. One of the participants described the intergenerational impact from the ‘eha inflicted by the Kāne in her family, “*My great-grandfather was a very violent person. He was Hawaiian. So, I can’t imagine what he had seen as a child, but those stories were never passed down to me… but from him, my grandfather was violent and then my mother became very violent to me… there is a long history of violence. I still feel like I have expressions of that violence towards me through intimate partners. So even though I’m not a violent person and I don’t go around making trouble… I still feel like there’s that black streak… and that’s how we process anger and hurt*”.

In addition, many participants described instances of sexual violence. They shared mo‘olelo of themselves or of a Wahine in their family being sexually assaulted and/or raped by Kāne. In these occurrences, there was a common feeling of hopelessness. Many felt unprotected by members of their family who either committed the ‘eha, ignored the truth, or brought home their perpetrators. One participant described being sexualized and assaulted as a young girl, “*around 13, my body started changing and I used to get touched in my sleep… it used to be like dead hot in the middle of the summer and I was sleeping in layers of clothes and blankets so that I wouldn’t feel anything. But it didn’t stop anything from happening. Home is supposed to feel safe, but that wasn’t the case*”. In general, participants felt unprotected in their families and by the justice system from personal experience or the experience of others who were not believed. As mentioned in institutional ‘eha, participants believed perpetrators were more protected than them, resulting in feelings of inferiority. They attributed this ‘eha as gendered and connected to historical trauma.

Others expressed that their families’ inability to heal from their own experiences with violence, which caused ripple effects for them. One participant self-harmed because her father died by suicide, “*my dad committed suicide… he had some historical trauma and intergenerational trauma in itself… I ended up cutting myself. I was in intermediate. My teacher actually seen my wrist and dragged me to the counselor’s office*”. Many described mental health care to be stigmatized in their communities; therefore, ‘eha was transferred to them through unresolved mental health issues.

#### 3.3.2. Settlers and Hawaiians Influenced by Settler Colonialism

Participants reported being ridiculed and criticized for their lack of Hawaiian cultural identity by other Hawaiians and non-Hawaiians. Many reported not feeling Hawaiian enough and judged harshly by others, including Hawaiians involved in aloha ‘āina movements. One participant shared, “*my brother wanted us to go to the Makahiki (a celebration dedicated to the god Lono to commence a time of rest and the rainy season) [on Kaho*‘*olawe]. So, we went. And we were getting ready to be in the procession and then this one lady, she looks at me and my sister and she says,* ‘*oh, they Hawaiian or what?* ‘*… I questioned myself. What does it take to be Hawaiian enough?… to be a part of this ceremony? That was an experience that has really stayed with me. And it was a Hawaiian woman that said it!*” 

Feelings of frustration also came up as participants reported encountering cultural appropriation (inappropriate adoption of culture by members of another, typically more dominant people), while many participants and their family members experienced ostracization from practicing Hawaiian culture or being Hawaiian. Therefore, participants expressed outrage when witnessing non-Hawaiian people using Hawaiian culture for their own healing and capital gain. They also acknowledged that institutions, especially educational institutions, allow for the appropriation and the degradation of Hawaiian or Indigenous peoples in their curriculum. “*There is this de-value of Hawaiian women and what they can do and how strong they are and resilient they are at home. And then I went to [school in] New Mexico, and… the things that I thought was ugly and scary was beautiful to them because it spoke to my resilience. But it’s just what we [Wāhine] have done to survive. But then you have all these fucking hippie white women culturally appropriating everybody else’s cultures because they wanted to be liberated and it’s like fuck, you don’t know what it is to fight for liberation. Just because you burn sage, or you don’t wear a bra or hold signs on the side of the street don’t mean you know what it is to suffer. Try an entire like frikin 70 something years of just being you and surviving and not getting credit of any of that*”. Overall, personally mediated ‘eha came from within and outside participants’ own communities, cultures, and genders.

### 3.4. Theme 4: Internalized ‘Eha

Internalized ‘eha describes participants accepting limitations based on other’s stigmatizations. At this level, ‘eha was found to be detrimental to participants as it weakened their pride and self-worth in their identities as Hawaiian women.

#### 3.4.1. Internalized ‘Eha from Suppressed Language and Culture

The institutional ‘eha of suppressing language and culture trickled down to internalized feelings of uncertainty and self-doubt, particularly regarding cultural identity. Many struggled with feelings of uncertainty and self-doubt about being Hawaiian, often feeling alienated or intimidated by others who were more familiar with Hawaiian culture. This internalized pain affected their confidence and sense of belonging in the Hawaiian community. Some seemed to accept their contention with their identities, which resulted in less engagement with their culture because they felt like they did not know enough. “*When I was growing up, we didn’t even we didn’t even learn Hawaiian history and it was hard for me to really, really claim being Hawaiian. I was a legal secretary, and I worked for [prominent Hawaiian politician]. And at that time, she was the [leader] of [a Hawaiian movement]. And I had to type the bylaws, and I’m typing it, and I never really joined the [movement]. Because I was so confused… I was just so so torn, and even though [the prominent politician] was one of my best friends, it was hard for me to actually delve into the whole movement*”. They also reported feeling intimidated by other Hawaiians due to a perceived lack of understanding of Hawaiian culture, which has contributed to a strain on their confidence and their sense of identity as Hawaiians. One participant expressed, “*so sometimes I do feel intimidated you know around other Hawaiian people. Cause I don’t know, I don’t know anything, and it sucks*”. 

#### 3.4.2. Internalized ‘Eha from Domestic Violence

Participants shared experiences of witnessing or enduring domestic violence, particularly in patriarchal family structures. These experiences reinforced Westernized gender roles and expectations, leading to further emotional and psychological damage. Women often endured mental, physical, and emotional violence in order to maintain family dynamics, perpetuating unhealthy patterns across generations. Through mental, physical, and emotional violence, Wāhine in these stories reinforced patriarchal structures in order to survive. “*My dad could degrade her and she’s gonna sit there and take it, and then ask him if he wants her to make his fucking plate… and when it comes to money, because she wants my dad to have that pride… he’s the breadmaker even though she got a fucking raise, but she can’t tell him because she wants him to feel really good about himself. And that was the same for her mom, my grandma… get beat up and [ask my grandpa] what you like for dinner? When we were younger, my dad actually didn’t even have a job and he was still the man of the house. Jobless for years, but he was still taking credit*”.

#### 3.4.3. Internalized ‘Eha from Intergenerational Trauma

Participants also reflected on the cyclical nature of trauma, where the pain they internalized was passed down to subsequent generations. They acknowledged how their own experiences with trauma, particularly linked to substance abuse, domestic violence, and lack of resources, became part of a broader pattern of dysfunction that impacted their children. Many expressed a sense of inevitability in passing on this trauma, despite their desire to break the cycle. One participant shared, “*Yeah, and I feel bad because I don’t want that for my son but that’s exactly what happens now… I always tell myself I don’t want that for him, I know how it feels but it’s just natural. It just comes and…in hindsight I’m like frick I should do it this way, but then the next time comes and it just happens*”. 

Many participants brought up health and social behavioral issues that impacted them. Substance misuse and domestic violence were commonly stated by participants as ‘eha that deeply affected their self-esteem, mental health, and the choices they made throughout their lives. “*My grandmother had her child. And she became a single parent from all these things like substance misuse, lack of education, domestic violence, but what happened is that then my mother had her first child and again, single parent. And then it comes down to me and I had my child at a very young age and was a single parent. It repeated itself through generations*”.

Overall, internalized ‘eha was found to impact individual well-being in addition to perpetuating cycles of trauma and cultural disconnection. Participants’ stories highlighted the complex relationship between cultural suppression, domestic violence, and intergenerational trauma and revealed that these factors shaped self-identity, self-esteem, and generational patterns of suffering.

## 4. Discussion

The overall purpose of this research project was to understand the historical trauma experiences of Wāhine. In this project, historical trauma was conceptualized as ‘eha. The findings reveal that Wāhine experience ‘eha at multiple levels—structurally, institutionally, interpersonally, and internally—and that these experiences are shaped by both ethnic and gender identities. While Wāhine’s experiences of ‘eha align with those of other Hawaiians across genders, they also face gendered forms of violence and oppression that are unique to their identities as women. These findings align well with previous Indigenous research describing gendered experiences of historical and intergenerational trauma [18,19,56]. In essence, Wāhine are oppressed and experience ‘eha through both their ethnic and gender identities through Westernized systems that oppress and dismiss their identities and mana simultaneously as Hawaiians and as women. Generally, participants readily identified colonialism (which is contemporarily upheld through structural and institutional ‘eha) as a key factor in creating these forms of ‘eha. The unresolved ‘eha from kūpuna who lacked access to healing, has transmitted to subsequent generations, which is reinforced by the sense of inferiority and ‘eha inflicted by Kāne and Western institutions and cultural norms.

The findings of this research highlight the deep and multifaceted nature of historical trauma, specifically focusing on the violence and oppression experienced by Wāhine, which has been inherited from previous generations through colonialism, patriarchy, and capitalism. As participants in this research project, the trauma and violence resulting from these systems not only impacted them but also affected their kūpuna (ancestors) and continues to reverberate through generations. The overthrow of the Hawaiian Kingdom and the subsequent imposition of Westernized gender roles have been identified as key events that shaped the current lived realities of Hawaiian women. These findings align with existing critiques of Native Hawaiian scholars, who have pointed out the transformation of Hawaiian sovereignty, culture, and understandings of mana through colonial political actions that militarized and commodified Hawai’i’s land, people, and culture [57,58,59,60,61,62]. Consequently, Hawai’i, along with its resources and its people, was rendered subordinate to Western agendas, a process that has had long-lasting implications on the health, well-being, and identity of Hawaiians, particularly Wāhine. This historical backdrop has resulted in ongoing social and health disparities within Hawaiian communities, as documented in the literature [58,59,60,61,62,63].

In addition to experiencing violence stemming from colonialism, participants in this project described what has been termed “double colonization”—being oppressed both by external structures (such as colonial powers) and by Kāne (men) within their families and communities [24]. This dual form of oppression is crucial to understanding the specific ways in which Wāhine face compounded forms of violence and discrimination. While much research on historical and intergenerational trauma points to colonization as the root cause of Indigenous oppression, this project emphasized the need to broaden the lens to fully understand the distinct experiences of Wāhine. As Indigenous scholar, Val Kalei Kanuha [1], aptly stated, the myth that colonization alone is the root of violence against women overlooks the gendered dimensions of oppression. Hawaiian women, though colonized, do not perpetuate violence against men at the same rate as their male counterparts perpetrate violence against them. This distinction underscores the need to consider how various “isms”—such as sexism, racism, classism, and homophobia—intersect to perpetuate ‘eha against Wāhine.

Sexual and domestic violence, particularly perpetrated by Kāne, was generated as a significant theme in participants’ narratives. This qualitative finding is consistent with existing data on violence against Hawaiian women. Studies show that Wāhine experience higher rates of intimate partner violence compared to non-Hawaiians and the general population in Hawai’i [63]. Furthermore, a study of sex trafficking victims revealed that 64% of those affected were Hawaiian, further illustrating the vulnerability of Wāhine to various forms of violence [64,65]. These statistics are a stark reminder of the systemic oppression that continues to shape the lives of Hawaiian women, and they underscore the urgency of addressing these issues at both local and global levels.

The recent cases of missing and murdered Indigenous women, including those of Ariel Sellers, a 6-year-old girl allegedly abused and murdered by her adoptive parents [65], and the ongoing disappearances of Hawaiian women, highlight the urgent need for systemic change [64]. Such cases not only exemplify the risks of sexual and domestic violence that Wāhine face, but also expose the failure of institutions to protect them. The introduction of Ariel’s Bill (HB 2424)—which aimed to allocate additional resources to the child welfare system—presented a potential step toward accountability, but the bill was vetoed by Governor David Ige in 2022, which illustrates the institutional inertia that continues to allow and sustain violence against Indigenous girls and women [66,67]. This decision underscores the need for institutions of power, including government and legislative bodies, to be held accountable for their role in perpetuating violence against Indigenous women.

The impact of colonialism is further compounded by the cultural erasure and marginalization that Wāhine face. Many participants expressed concern about policies that extract Hawaiians from their culture and land, which they identified as both a symptom and cause of the violence they experience. The loss of cultural identity, often attributed to the violence of Kāne, contributes to feelings of insecurity and inferiority, creating a cycle of ‘eha. Research indicates that a strong sense of cultural identity is a protective factor against poor health outcomes, suggesting that reclaiming cultural identity could be a key component in breaking this cycle of violence and trauma [4,58,66,68,69,70]. Moreover, frameworks like the Social Determinants of Health emphasize that health outcomes are shaped not only by individual behaviors but by broader social, political, and economic conditions [69]. These frameworks reinforce the notion that addressing the ’eha experienced by Wāhine requires a multi-level, systemic approach that holds every sector and institution accountable for their role in perpetuating gender violence and inequities.

### 4.1. Addressing Wāhine ‘Eha

While public health programs often focus on building resilience in marginalized groups [70], it is insufficient to place the responsibility of healing solely on Wāhine. A true systemic approach to healing would redistribute the ownership of ‘eha, making the entire system responsible for Wāhine health. It is essential to recognize that healing does not rest on individual resilience alone; rather, it requires an acknowledgment of the broader social and institutional forces that continue to perpetuate violence and trauma.

One promising approach to addressing this issue is the Protect Maunakea Movement [71], which has been integral in promoting shared leadership between Wāhine and Kāne. This approach challenges patriarchial gendered leadership structures and reflects the importance of inclusivity and gender fluidity in decision making. Similar to efforts by Black, Indigenous, and People of Color (BIPOC) communities in the continental U.S., diversifying leadership within various sectors can increase opportunities for equity and healing [72,73]. However, simply having women in leadership roles is not enough. Leaders must be pono (righteous) and committed to values like aloha ‘āina (love for the land)—values that are integral to Hawaiian well-being.

Incorporating aloha ‘āina into educational curricula and public policies is another critical aspect of promoting the well-being of Wāhine. Hawaiian culture, language, and history must be represented across all educational settings, not just within Hawaiian language schools or Kaiapuni (Hawaiian immersion charter schools). This ensures that all learners, from children to adults, understand their kuleana (responsibility) to Hawai’i and her people, counteracting the erasure of Hawaiian identity that has resulted from colonization. The University of Hawai’i at Mānoa’s requirement for undergraduate students to take courses on Native Hawaiian perspectives is one example of how institutions can help foster a sense of cultural identity and responsibility [74]. However, universities and other institutions must also critically examine how they perpetuate ‘eha, particularly in their treatment of Wāhine given the high rate of gender violence reported by college students.

The need to address violence and trauma against Wāhine extends beyond education and includes institutions such as the U.S. military, tourism industry, criminal justice system, and mental health services. Participants in this research project highlighted the role these systems play in perpetuating the cycle of violence and inequity. Addressing ‘eha within these systems requires a collective and intersectional approach that ensures Wāhine are neither erased nor invisible.

Ultimately, the liberation of Wāhine is inextricably linked to the liberation of all Hawaiians, from keiki (children) to kūpuna (elders). Healing, therefore, must be collective. A systemic approach to healing would focus on restoring the ‘ohana (family) unit, kaiaulu (community), and the Lāhui (Hawaiian nation). Frameworks such as Kūkulu Kumuhana illustrate that health and well-being are optimal when every level of society—from the family to the broader community—embraces values such as ‘āina momona (fertile land), ea (self-determination), and pilina (relationships) [75]. As demonstrated by health programs like Mokuau, Braun, and Daniggelis’ [76] work to help Wāhine recover from breast cancer, healing is relational and collective. Systems, including the criminal justice system, should be reimagined with a collective approach to benefit Wāhine, and further research should explore what that might look like.

### 4.2. Limitations and Strengths

The findings of this project are not without limitations. Further research is needed to expand our understanding of how historical trauma is transmitted and how it manifests in different generations. Future studies should focus on multigenerational families to explore the transmission of trauma and to identify targeted interventions that can address the specific needs of Wāhine and their communities. Additionally, future research could employ visual methods, such as lifeline interviews, to chart trauma across time and generations [77,78].

One of the strengths of this project lies in its thoughtful and purposive sampling approach, which was particularly important given the sensitive nature of trauma and violence. Although obtaining a larger sample size proved challenging due to the emotional weight of the topics discussed, the research reached theoretical saturation with the number of interviews conducted. This suggests that the sample size was sufficient to provide rich, meaningful insights into the experiences of Wāhine, allowing for a robust analysis of the data.

Another key strength of this project is its alignment with Indigenous methodologies, which emphasize relational and culturally grounded approaches to knowledge production [79,80]. Participants in this project had an existing relationship with the researchers, which facilitated an environment of trust and mutual respect. While conventional Western research might view this prior relationship as a potential source of bias, in this context, it was considered to be an asset. The existing trust and genuine relationships between the researcher and participants enabled more in-depth storytelling and allowed participants to share their traumatic experiences in a safe and supportive environment. This relational foundation was essential for obtaining rich, personal narratives that deepened the understanding of the complex ways in which trauma and violence manifest in the lives of Hawaiian women. The interconnection between the researcher’s existing relationships and the Indigenous lens of the research team also contributed to a more nuanced and comprehensive analysis of the data.

Additionally, the research project took care to approach the complex and difficult nature of trauma and violence with the necessary caution and care. The researcher’s personal connection to the community and the emotional labor that comes with conducting research in one’s own community was both a strength and a challenge. When Indigenous researchers approach research within their communities and for their communities, a heavy burden of kuleana is placed on both of these aspects, individually and collectively. Indigenous researchers are not just demystifying issues to ensure the overall well-being of the public, they are also demystifying personal issues and those that directly impact their families and communities. Therefore, in this project, data collection took longer than expected. However, the time taken to ensure thoughtful data collection and analysis reflects the care and responsibility inherent in Indigenous research practices. Indigenous researchers studying with and for Indigenous communities ensure that the research is conducted with sensitivity to the emotional and cultural complexities involved.

In sum, the strength of this project lies in its Indigenous methodological framework, the relational approach to data collection, and the careful attention given to both participant and researcher well-being. Through its thoughtful design and community-centered approach, the findings of this project offered valuable insights into the lasting impacts of violence and trauma, while also laying the groundwork for future research aimed at healing and justice for Hawaiian women.

### 4.3. Future Research

While this study offers a comprehensive detailed experiences of ‘eha, it also points to areas for future research, particularly in exploring the transmission of trauma across generations. Utilizing a lifeline method for future studies is particularly valuable, as it provides a visual tool for tracking life histories and understanding how traumatic experiences are passed down within families [77,78]. By comparing the lifelines of grandmothers, mothers, and daughters, future research can deepen our understanding of the intergenerational impacts of trauma and identify specific points of intervention that could promote healing. This approach holds promise for informing targeted strategies that can better support the health and well-being of Wāhine in the future.

## 5. Conclusions

Addressing the accumulated ‘eha of Wāhine has the power to uplift all of Hawai’i if systems and institutions prioritize collectivism in healing. Findings from this research project communicate the urgency for change to heal Wāhine with radical aloha to support them in the reimagination of a world that is inclusive of their needs and mana. Further data, both qualitative and quantitative, must be collected to deepen our understanding of ‘eha and create solutions for the well-being of our Wāhine.

## Figures and Tables

**Table 1:** Interview questions and their related domains of interest.

Domain	Interview Questions
Mo’okūauhau	After the interviewers shared their mo’okūauhau, participants were asked: Do you mind sharing a bit about yourself and your mo’okūauhau?
Perceived historical loss	Can you tell me about a historical event in Hawai’i that has impacted an older woman in your family?
Trauma response	How do you think that historical event has impacted you or a woman in your family?
Intergenerational trauma transmission and experiences	Are there any historical policies or processes that affect your family today? Can you tell me about a time you were treated differently because you are a Hawaiian woman? What are some of the roles the women in your family take on? What are some of the roles the men in your family take on? Do you think the trauma of the past influenced/changed these roles? Can you tell me about a time you were treated unfairly by a man in your family, personal relationships, or outside of your family? Can you tell me about a time you felt excluded from a decision that really impacted you as a woman? Who do you think is responsible for the trauma or wrongdoings that have happened to your ‘ohana? What do you hope to see with future generations to end the cycle of intergenerational trauma?

**Table 2:** Characteristics of key informant interview participants.

Characteristics	Values *n* (%)
Ages	
Kūpuna (60+)	3 (23%)
Mākua (25–59)	7 (54%)
‘Ōpio (18–25)	3 (23%)
Marital status	
Single	5 (38%)
In a relationship or married	3 (23%)
Divorced, separated, or widowed	5 (38%)
Geographical location	
Rural	9 (69%)
Urban	4 (31%)
Have children	11 (85%)

## Data Availability

The stories and data that are shared in this research project belong to individuals who are represented in this paper.
